# Inadequate reporting of sample size calculations in cluster randomised trials: a review

**DOI:** 10.1186/1745-6215-14-S1-P122

**Published:** 2013-11-29

**Authors:** Clare Rutterford, Monica Taljaard, Stephanie Dixon, Andrew Copas, Sandra Eldridge

**Affiliations:** 1 Queen Mary University of London, London, UK; 2 Ottawa Hospital Research Institute, Ottawa Hospital, Ottawa, Canada; 3 Schulich School of Medicine and Dentistry, University of Western Ontario, London, Canada; 4 MRC London Hub for Trials Methodology Research, London, UK

## Objectives

To assess the adequacy of reporting sample size calculations in published cluster randomised trials (CRTs) and to evaluate the accuracy and justifications behind the a priori estimates used.

## Methods

A review was conducted of 166 CRTs reporting sample size calculations published between 2000 and 2008. Each trial was reviewed independently by two statisticians. The adequacy of the reporting of key elements in the CONSORT recommendations for CRTs was evaluated. Comparisons were made between the authors' a priori assumptions and values then observed in the trial.

## Results

Of 166 trials, only 56 (34%) reported all key elements of sample size calculations in line with CONSORT recommendations. Elements specific to CRTs were the worst reported: the number of clusters or average cluster size was specified in only 94 (57%) and a measure of intracluster correlation coefficient (ICC) in only 86 (52%). Only 20 papers (12%) reported a priori and observed ICC values. In the majority of these reports, the a priori estimate for the ICC was conservative compared to the observed value. Few authors provided justifications for their choice of a priori estimates. Not unexpectedly, trials which reported no statistically significant difference were more likely to observe effect sizes smaller than the assumed clinically important difference.

## Conclusions

Even with the CONSORT extension to CRTs, the reporting of sample size calculations in CRTs remains below that necessary for transparent reporting. Further awareness is needed to encourage the reporting of observed ICCs in order to evaluate the choice of a priori estimates and interpret the trial results.

